# Keratan Sulfate: An Electroconductive Glycosaminoglycan at the Interface of Sensory Perception and Neural Signaling

**DOI:** 10.1002/jnr.70149

**Published:** 2026-07-31

**Authors:** James Melrose

**Affiliations:** ^1^ Raymond Purves Lab, Kolling Institute, Faculty of Medicine and Health University of Sydney and Northern Sydney Local Health District, Royal North Shore Hospital Sydney New South Wales Australia; ^2^ Graduate School of Biomedical Engineering, Faculty of Engineering University of New South Wales Sydney New South Wales Australia

## Abstract

This narrative review highlights the functional attributes of keratan sulfate (KS), a glycosaminoglycan integral to structural support, hydration, and diverse tissue functions. KS has roles in cell signaling and regulates cellular adhesion, proliferation, and differentiation. The electroconductive properties of KS stabilize ion fluxes and electrochemical gradients at the cell surface essential for membrane polarization that directs neuronal activation and neurotransmission. KS interacts with a diverse collection of kinases, growth factors, morphogens, and neuro‐modulatory proteins, facilitating axonal guidance and correctly inter‐connected neural networks essential for control of tissue function. The ability of KS to capture and transport protons assists in neuronal signaling and provides an ultrasensitive system that allows neurons to participate in neurosensory processes and contribute to neurosensory perception. The roles of some KS‐proteoglycans are highlighted and encourage further studies in innovative areas of sensory perception and tissue function.

## Significance Statement

Understanding how neurons activate and contribute to cell signaling during neurotransduction is essential to brain function. Keratan sulfate has electroconductive, proton‐capturing, and proton‐transporting properties that may support neurotransmission, making it a potentially important neural interface molecule for future neural repair strategies. With neurological disorders affecting nearly one billion people worldwide, innovative neuroregenerative approaches are urgently needed.

## Graphical abstract

Laboratory studies show KS has electroconductive properties with the ability to trap and transport protons in neurotransductive processes (Grotthuss proton shuttling). This maintains electrochemical gradients and fluxes at the neuronal cell surface in neural activation. Such processes are central to neuronal cell signaling and communication.

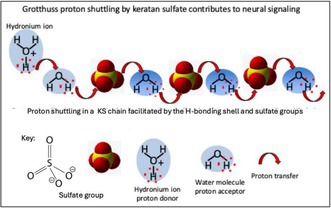

## 1 Introduction

**FIGURE 1:**
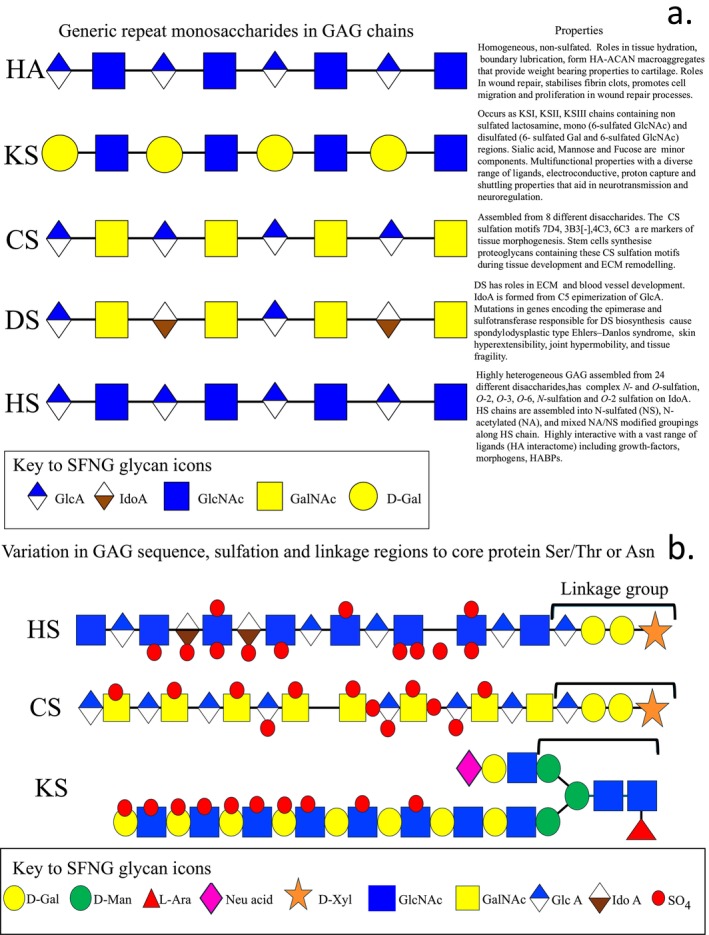
Schematic depiction of GAG structure showing their generic repeat disaccharides and functional properties (a) and variations in their sulfation patterns and linkage structures to proteoglycan core proteins (b). The structures are delineated using SFNG glycan icons (Varki et al. 2015; Neelamegham et al. 2019).

Glycosaminoglycans (GAGs) are important components of the extracellular matrix (ECM) and are also interactive cellular components. Five GAGs have been identified on the basis of their repeat disaccharides and differing structures in their linkage regions to proteoglycan (PG) core proteins. These include hyaluronan (HA), the only non‐sulfated GAG; heparan sulfate (HS); chondroitin and dermatan sulfate (CS, DS); and keratan sulfate (KS), each with distinct functional properties in a range of tissues (Figure 1). GAGs are attached to serine or threonine through *O*‐linkages or to asparagine through *N*‐linkages to proteoglycan (PG) core proteins.

## 2 Evolution of GAGs in Early Life Processes

During the early evolution of life, GAGs evolved under strict evolutionary selection criteria as glycocalyx components surrounding all cells with properties of molecular recognition, information storage, and transfer properties that provided cell instructive properties. This property was encoded in the remarkable properties of the glyco‐code carried by glycan structures; this is a sophisticated instructional and information storage cell instructive system rivaling the information encoded by DNA/RNA (Gabius 2015). Hyaluronan emerged relatively late in evolution, possibly to evade immune recognition (Csoka and Stern 2013). Chondroitin sulfate is a more ancient GAG than HA; epimerization of 4‐hydroxyl groups in GalNAc of CS to GlcNAc in HA is the only structural difference between CS and HA, other than the relative chain lengths of these GAGs. The exposed CS axial 4‐hydroxyl group in GalNAc extends out in the equatorial plane and may be a prime target for immune detection. This also shows the important roles glycosyltransferases (Kikuchi and Narimatsu 2006) played during this evolutionary period, which facilitated modifications in GAG structure (Lairson et al. 2008; Breton et al. 2012). Loss of ancestral N‐glycosylation sites in conserved proteins during human evolution also impacted the evolution of protein structure (Breton et al. 2012).

HS is an ancient molecule of the glycocalyx that evolved as a molecular recognition cell regulatory molecule early in metazoan evolution (Yamada et al. 2007, 2011) and in a 500 million year period became a molecule with immense molecular diversity (Esko and Lindahl 2001) acting as a molecular recognition and information storage molecule with cell interactive properties which allowed it to control cellular behavior (Esko and Selleck 2002). KS is a molecular signature of medullary bone and has been detected in fossilized dinosaur bones (Canoville et al. 2021) showing its ancient evolutionary origins. Dinosaurs first appeared in the Triassic period around 245 million years ago.

### 2.1 The Sophisticated Properties of GAGs in Tissue Development and Function

Dermatan sulfate and CS promote assembly of the ECM mediated by cellular interactions with growth factors to promote cell proliferation and differentiation and tissue morphogenesis during development (Chen et al. 2025). Proteoglycans decorated with CS and DS side chains have interactive properties with collagen fibrils and have instructive properties that direct axonal migration and formation of neural networks in CNS/PNS development (Mizumoto and Yamada 2022; Mizumoto et al. 2022; Schwartz and Domowicz 2023). DS has crucial roles in the development of blood vessels, bone, and the CNS/PNS (Mizumoto and Yamada 2022; Mizumoto et al. 2022; Schwartz and Domowicz 2023). CS/DS glycotransferase and sulfatase biosynthetic enzymes have decisive roles in tissue development (Habicher et al. 2022). This is exemplified in mutations in human genes that encode epimerase and sulfotransferase DS biosynthetic enzymes; these cause connective tissue disorders including spondylodysplastic type Ehlers–Danlos syndrome, resulting in skin hyperextensibility, joint hypermobility, and tissue fragility (Mikami et al. 2025). DS‐deficient mice show perinatal lethality, skin fragility, vascular abnormalities, thoracic kyphosis, dysfunctional muscle developmental phenotypes, and impaired self‐renewal and proliferation of neural stem cells (Mizumoto and Yamada 2022). CS and DS have important instructive properties over neural cell populations that direct developmental processes in the CNS/PNS (Chen et al. 2025) and essential roles in the maintenance of neural tissue homeostasis and repair processes in traumatized neural tissues (Hayes and Melrose 2021; Siddiqui et al. 2022).

The sulfation motifs on CS are important functional determinants and necessary for proper Indian hedgehog signaling (Cortes et al. 2009). However CS‐PGs have also been identified that can inhibit IHH (Takemura et al. 2020). Windpipe (Wdp) is a novel CS‐PG in *Drosophila*. Wdp is a single‐pass transmembrane protein with leucine‐rich repeat (LRR) motifs and bears three extracellular CS chains Wdp modulates the Hedgehog (Hh) pathway. In the wing disc, overexpression of *wdp* inhibits Hh signaling, this is dependent both on its CS chains and its LRR motifs.

HS‐PGs and CS‐PGs show opposing effects in neural systems; axon growth is typically promoted by HSPGs but inhibited by CSPGs (Bandtlow and Zimmermann 2000; Kantor et al. 2004; Silver and Miller 2004; Cregg et al. 2014; Van Vactor et al. 2006; Matsumoto et al. 2007; Coles et al. 2011). Dual interaction of HS and CS with Receptor Like Protein Tyrosine Phosphatase‐sigma (RPTPσ) may represent a molecular switch that regulates neural clustering and neuronal extension in the development of neural networks (Coles et al. 2011) and tissue development in health and disease (Xu and Fisher 2012).

### 2.2 The Three KS Isoforms Occurring in Tissues

**FIGURE 2:**
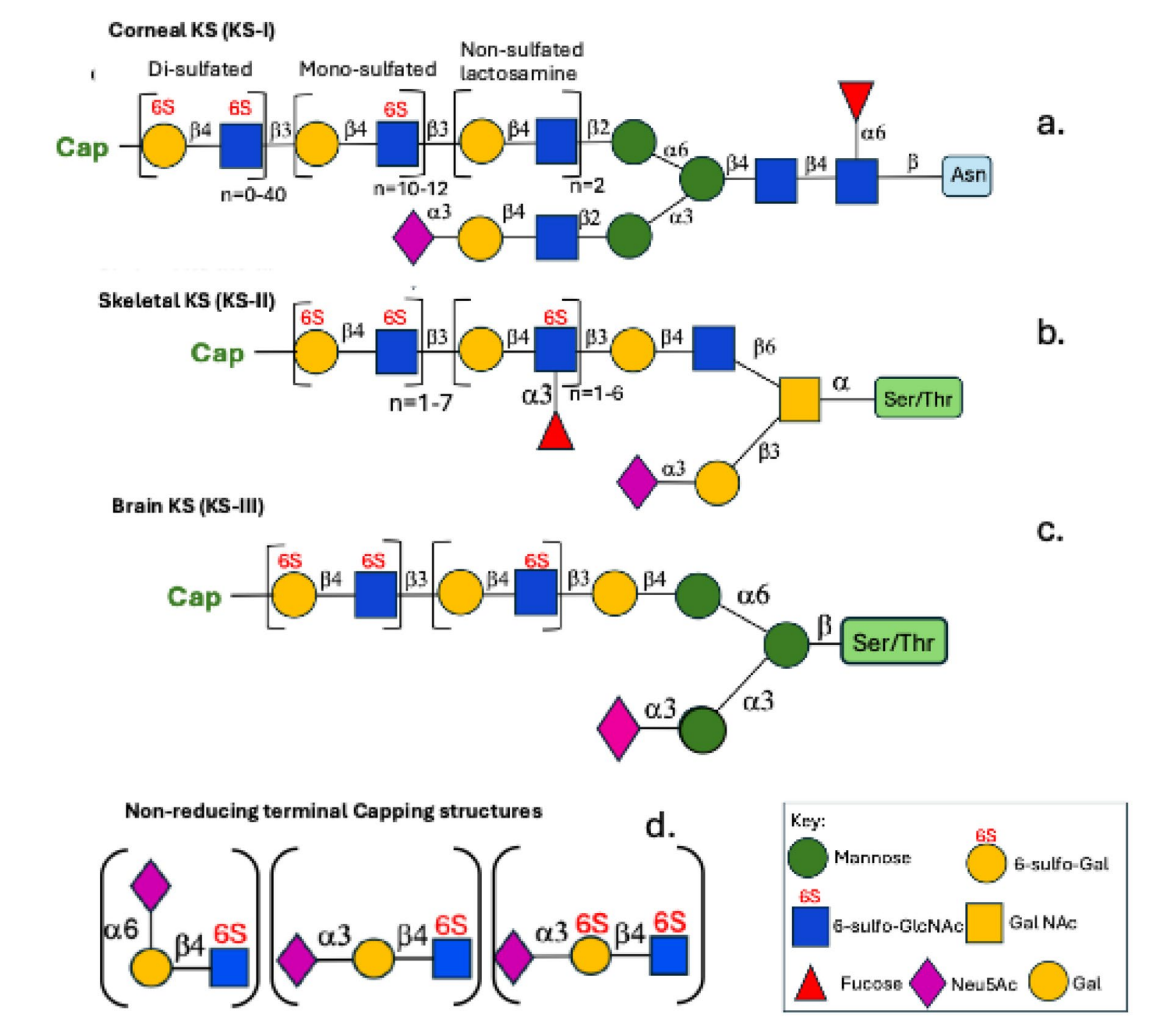
A schematic overview of corneal KS‐I (a), skeletal KS‐II (b), and brain KS‐III (c) using SFNG glycan icon nomenclature (Varki et al. 2015; Neelamegham et al. 2019). It highlights regions of variable sulfation, including non‐reducing terminal disulfation, internal mono‐sulfation, and non‐sulfated lactosamine. The diagram also depicts variable non‐reducing terminal capping structures (d) and both *N*‐linked and *O*‐linked linkage regions to proteoglycan core proteins. Notably, brain KS chains can be either *N*‐linked or *O*‐linked to proteoglycan core proteins via asparagine and serine/threonine amino acid residues respectively, depending on proteoglycan and tissue niche context. Brain aggrecan contains *O*‐linked KS, whereas lumican, keratocan, and fibromodulin contain *N*‐linked KS. Small *N*‐ and *O*‐linked KS chains also occur in the N terminal G1 and G2 globular domains of aggrecan and in the inter‐globular region between them.

**FIGURE 3:**
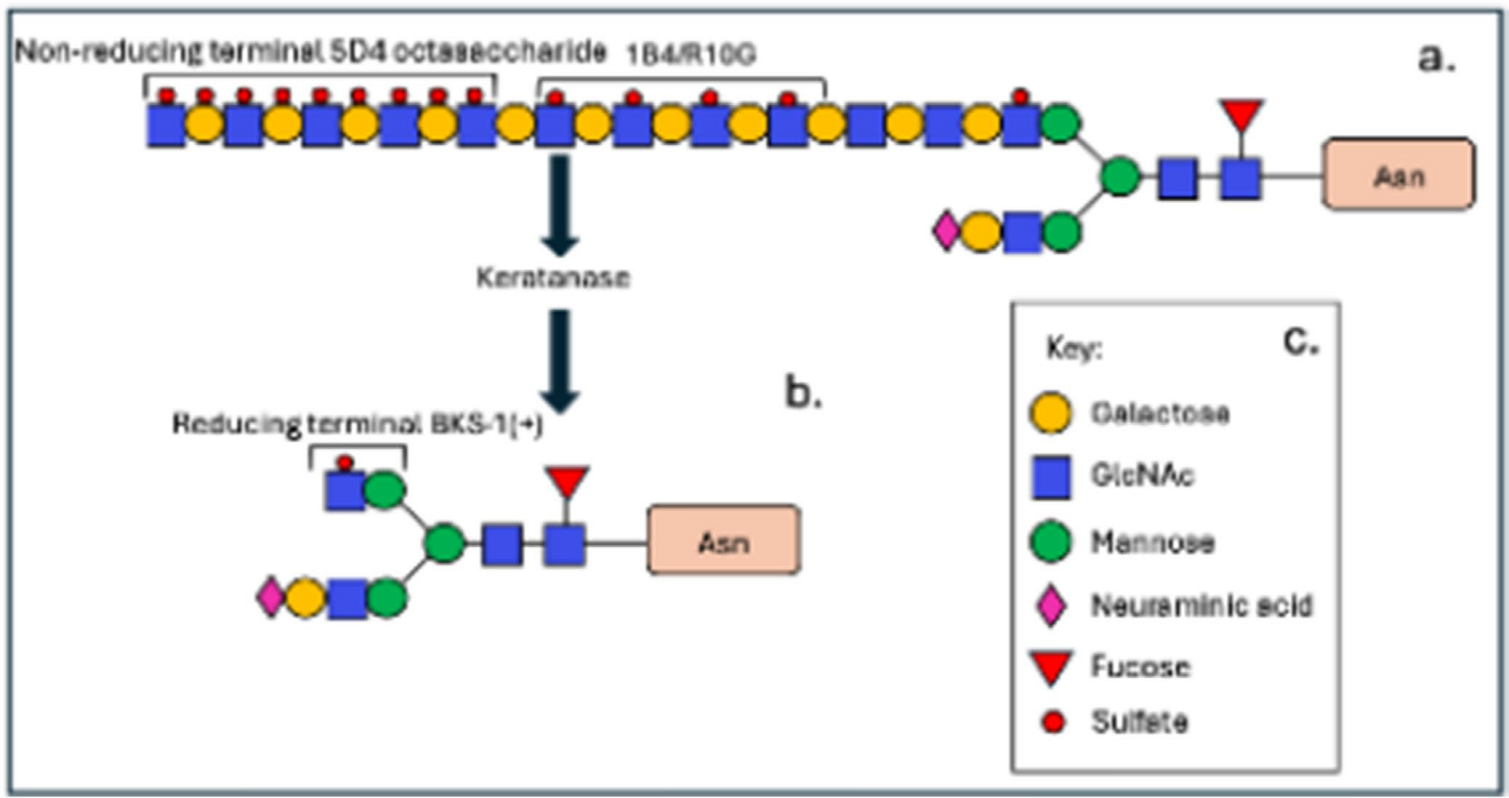
Schematic depiction of high and low sulfation regions in KS including the 5D4 octasaccharide, 1B4/R10G low sulfation epitopes on corneal KS‐I (a) and the keratanase generated BKS‐1 (+) neoepitope in the reducing terminus (b) and a description of the SFNG icons for glycan residues used in this schematic (c). BKS‐1 (+) is a reducing terminal N‐acetyl glucosamine‐6‐sulfate neoepitope.

Keratan sulfate (KS) is a highly specialized GAG that represents a sophisticated, relatively recent evolutionary development in vertebrates critical for the function of specialized tissues like the cornea, brain, and cartilage. Three forms of KS have been identified (Figure 2) (Caterson and Melrose 2018). KS chains have regions that are disulfated on Gal and GlcNAc towards the non‐reducing terminus, are mono sulfated on GalNAc internally, and non‐sulfated in a lactosamine region adjacent to the reducing terminus (Tai et al. 1996). The repeat KS disaccharide in KS is β3Gal‐β1‐4GlcNAc‐β1‐. Three forms of KS have been identified: Corneal (KS‐I), cartilage (KS‐II), and brain (KS‐III); these have differing linkage regions which attach them to proteoglycan core proteins (Seno et al. 1965). KS‐I can be *O*‐linked to Ser/Thr or *N*‐linked to Asn residues, KS‐II is *O*‐linked to Ser/Thr in the core protein of brain aggrecan (Krusius et al. 1986), and KS‐III is attached to core proteins of lumican, keratocan, mimecan, fibromodulin, PRELP, and osteoadherin *N*‐linked to Asn (J. Funderburgh 2000). KS‐II is highly sulfated, with disulfated regions predominating over monosulfated regions (Nieduszynski et al. 1990). The sulfation motif on KS is an important functional determinant which determines its interactive properties (Caterson and Melrose 2018). Spatiotemporally expressed glycosyl and sulfotransferases in tissues determine KS functional diversity (Habuchi 2014; Yasuoka 2023). High and low sulfation KS (Figure 3) have cell instructional roles in the CNS and a range of other tissues (Melrose 2025a, 2024a). A number of antibodies are available to different regions of the KS chain and to specific epitopes, aiding in analysis of the complex roles of this GAG (Caterson and Melrose 2018) and also illustrate its structural complexity (Table 1).

**TABLE 1:** KS antibodies demonstrating its structural complexity.

Ab clone	Epitope recognized	References
R‐10G	Galβ1‐4GlcNAc(6S)β1‐3Galβ1‐4GlcNAc(6S)β1	Nakao et al. (2017)
R‐6C	Siaα2‐3Galβ1‐3GlcNAc(6S)β1‐3Galβ1‐4GlcNAc(6S)β1	Nakao et al. (2023)
R‐17F	Fucα1‐2Galβ1‐3GlcNAβ1‐3Galβ1‐4Glc (lacto‐N‐fucopentaose I)	Nakao et al. (2023)
R‐13E	Fucα1‐2Galβ1‐3GlcNAcβ1‐3Galβ1	Nakao et al. (2023)
BKS‐1(+)	GlcNAc (6S) keratanase‐1 generated stub on the linkage region	Hayes and Melrose (2020); Akhtar et al. (2008)
1‐B‐4	Monosulfated Galβ1‐4‐GlcNAc(6S) on tetra sulfated KS	Mehmet et al. (1986)
5‐D‐4	Gal(6S)β1‐4‐GlcNAc(6S) oversulphated octasacchide, strong binding to a minor disulphated Gal(6S)β1‐4‐GlcNAc(6S) dodecasaccharide	Caterson et al. (1983); Mehmet et al. (1986)
MZ15	Epitope specificity similar to that of MAb 5D4	Mehmet et al. (1986); Heath and Thorogood (1989); Thornton et al. (1989)
294‐1B1	2–3 tandem GlcNAc‐6‐*O*‐sulfated LacNAc repeat units	Hoshino et al. (2024)
3D12/H7	Novel KS domain in the CS region of human articular aggrecan	Fischer et al. (1996)

#### 2.2.1 A Diverse Range of Antibodies Have Identified Potential Functional Determinants in KS

KS chains can be *O*‐linked to serine or threonine residues or *N*‐linked to asparagine on PG core proteins. The D‐mannose linkage regions of the KS chains are branched structures; one of these KS chains does not undergo elongation and is prematurely truncated. The KS chain that undergoes elongation does so through addition of generic GlcNAc‐galactose repeat disaccharides; however, it is not uniformly sulfated and contains non‐sulfated polylactosamine, monosulphated GlcNAc, and dually 6‐sulfated GlcNAc and D‐galactose in the non‐reducing terminus. Historically, anti‐KS antibodies (5D4) were raised to these oversulfated regions in KS (Caterson et al. 1983; Mehmet et al. 1986) and it was only with the development of further antibodies (1B4, R‐10G) (Nakao et al. 2017) that the contributions of the lesser sulfated regions of the KS chains became apparent. Further development of antibodies that detect terminal sialic acid (R‐6C) and L‐Fucose (R‐13E, R‐17F) end capping structures on KS chains further demonstrates the complexity of KS; however, the function of these epitopes still has to be determined (Table 1). A neo‐epitope MAb, BKS‐1(+), has also been developed which detects a 6‐sulphated GlcNAc on a keratanase‐1 generated disaccharide stub epitope attached to the KS linkage region (Hayes and Melrose 2020).

### 2.3 Biosynthesis of KS

Synthesis of KS relies on the coordinated action of multiple glycosyl transferases that sequentially add Gal and GlcNAc residues to the growing KS chain followed by sulfotransferases that sulfate these residues at C6 but not in a uniform manner with bi‐, mono‐, and non‐sulfated regions in the KS chain evident. Extension of the Man linkage region of KS may result in branching from the main chain (Itoh et al. 2026). KS is the only branched GAG (Caterson and Melrose 2018) and is highly interactive with a diverse range of ligands (Conrad et al. 2010).

#### 2.3.1 KS Biosynthetic Enzymes

##### 2.3.1.1 *N*‐Acetylglucosaminyltransferases

Seven β3N‐acetyl glucoaminyltransferases (β3GnT‐1, 2, 3, 4, 5, 6, 7) catalyze the addition of GlcNAc via β3 linkage to non‐reducing terminal Gal or GalNAc (J. Funderburgh 2000, 2002).

##### 2.3.1.2 Sulfotransferases

Sulfotransferases transfer sulfate groups from 3′‐phosphoadenosine 5′‐phosphosulfate (PAPS) donor to C6 of Gal or GlcNAc in KS; KSGal6ST transfers sulfate groups to C6 of Gal, and GlcNAc6ST transfers sulfate to the C6 of GlcNAc (J. Funderburgh 2000, 2002). Five GlcNAc‐6‐ST genes have been identified. GlcNAc6ST‐5 sulfates nonreducing terminal GlcNAc. Sulfation of Gal in unsulfated KS disaccharides is much lower compared to disaccharides where GlcNAc is already sulfated.

### 2.4 The Diverse Interactive Properties of KS: Proteomic Analysis of Corneal KS

Proteomic analysis of corneal KS showed highly sulfated KS interacted with 217 microarray proteins, including 75 kinases, several membrane or secreted proteins, many cytoskeletal proteins, and many nerve function proteins (Conrad et al. 2010). Of 85 ECM nerve‐related epitopes, KS bound 40 proteins, including SLIT, 2 ROBOs, 9 EPHs, 8 Ephrins (EFNs), 8 semaphorins (SEMAs), and 2 nerve growth factor receptors. This is consistent with the regulatory roles of KS‐proteoglycans through interactions with neurotrophic factors and neuroregulatory ligands (Melrose 2019a).

Layer specific proteomic analysis of cornea identified 4454 proteins demonstrating significant complexity but also provided insights into the structure and biological function of different regions of the cornea including the epithelium, endothelium, and sub epithelial region with several proteins identified with roles in wound healing and functional specialization of corneal regions (Schadwinkel et al. 2026).

#### 2.4.1 KS Sulfation Is a Critical Determinant of Ligand Interactivity

Sulfation motifs on GAGs are known functional determinants that facilitate interactions with a diverse range of ligands (Melrose 2019b). The subtle variation in the structure of KS may facilitate diverse roles undertaken by KS‐PGs not only in tissue development (Melrose 2019a) but also in neural interactions in the CNS/PNS (Melrose 2024a, 2025b, 2025c) (Table 2). Low sulfation KS isoforms have electroconductive properties in the gel filled Ampullae of Lorenzini sensory pores of elasmobranch fish species (Zhang et al. 2018; Josberger et al. 2016) and roles for low sulfation KS are emerging in mammalian tissues (Melrose 2025a, 2025b). The electrolocation detection system in sharks and rays is one of the most sensitive detection systems known in Nature. Sharks can detect electrical discharges as low as 5 nV/cm equivalent to the charge emitted by a flashlight battery to an electrode 1000 miles away (Hopkins 2026).

**TABLE 2:** KS‐proteoglycans found in the CNS/PNS and in cartilaginous tissues.

Proteoglycan	Core protein, Mw (kDa)	KS form	Proteoglycan properties	References
Phosphacan KS isoform	300	High sulfation KS	Soluble RPTP‐zeta ectodomain modulates neuritogenesis, PNNs	Faissner et al. (2006); Maurel et al. (1994); Garwood et al. (2003, 1999); Fujikawa et al. (2017)
Aggrecan ACAN	220–250	High sulfation KS region, *N*‐and *O*‐linked KS in G1, IGD, G2 domains	Forms PNNs, aggregates with HA hydrate and compartmentalize brain, HNK‐1 trisaccharide in aggrecan extends its cell interactive properties	Kiani et al. (2002); Morawski, Bruckner, et al. (2012); Yabuno et al. (2015)
SV2 SV2A, B, C isoforms	100, 250	High sulfation KS	Transmembrane synaptic vesicle transportation/storage of neurotransmitters	Nowack et al. (2010); Wan et al. (2010); Morgans et al. (2009); Dunn et al. (2017)
SLRPs Lumican LUM	38	N‐linked KS	LRR9 (lumcorin) MMP inhibitor, C‐terminal 13 amino acids (lumikine) growth factor, regulates corneal small collagen fiber 3D architecture, anti‐angiogenic, anti‐tumor activity	Nikitovic et al. (2008); Zeltz et al. (2009); Pietraszek et al. (2013, 2014); Puri et al. (2020)
Keratocan KERA	37–50	N‐linked KS	Developmentally regulated SLRP, aids in regulation of collagen fibrillogenesis in cornea and axonal migration	Puri et al. (2020); Gesteira et al. (2023); Matsushima et al. (2019); Stepp and Menko (2024)
Fibromodulin FMOD	42	N‐linked KS	Regulates large scleral collagen fibrillogenesis, roles in wound repair	Zheng et al. (2023); Jan et al. (2016); Al‐Qattan and Al‐Qattan (2018); Sjoberg et al. (2005); Svensson et al. (2000)
Podocalyxcin PODXL	65	Low/High sulfation KS isoforms	Sialo‐KSPG promotes neurogenesis and synapse stabilization. Low sulfation PODXL KS in embryonic development, high sulfation PODXL KS over‐expressed in many tumors	Le Tran et al. (2021); Toyoda et al. (2017); Vitureira et al. (2010, 2005)

Abbreviations: G1 and G2, N‐terminal globular domains; IGD, inter‐globular domain; LRR, leucine rich repeat; PNN, perineuronal net; RPTP‐zeta, receptor protein tyrosine phosphatase‐zeta; SLRP, small leucine rich repeat proteoglycan.

Corneal KS microarray and plasmon resonance analyses demonstrate interactions with 75 kinases, whereas low sulfation KS shows minimal binding (Conrad et al. 2010). Sulfation at the C6 position of galactose or GlcNAc modulates Eph receptor activity. In podocalyxcin, KS sulfation is low in normal embryonic cells but elevated in tumor cells, correlating with hyperactivation of MAPK and PI3K (Le Tran et al. 2021). During neural development, highly sulfated KS can inhibit axonal growth by clustering receptor tyrosine kinases, including Eph receptors. Conversely, reduced or absent KS sulfation, as observed in Macular corneal dystrophy and KS deficiency in corneal disorders such as keratoconus (Funderburgh et al. 1990) disrupts kinase signaling, suppresses Wnt activity, reduces MMP expression, and leads to abnormal stromal protein accumulation and impaired vision (Saito et al. 2008; Singh et al. 2021; Akama et al. 2000; Basol et al. 2025). Poorly sulfated KS is also less water soluble, impairing collagen fibril spacing resulting in corneal thinning and opaque macular deposits that compromise vision.

Sulfation dependent interactions between KS and Slit–Robo proteins activate Rho GTPases driving actin remodeling and cytoskeletal reorganization acting as molecular switches in axonal development (Delpech et al. 2024). KS binds IGFBP2 and regulates insulin dependent Cdk4 mediated neural proliferation and differentiation. KS functions as a co‐receptor presenting growth factors to kinases (Chirivella et al. 2017). KS‐Ephrin 4 receptor interactions promote neuronal cell proliferation and differentiation through Ephrin tyrosine kinase activity (Liu et al. 2017). Ephrins and Ephrin receptors are the largest subfamily of receptor protein tyrosine kinases. These are membrane‐bound proteins, which regulate intracellular signaling pathways arising from direct cell to cell interaction resulting in the activation of Rho family GTPases critical to axonal guidance in neural development. Axonal guidance proteins interactive with KS such as Slits contain variable numbers of LRR modules and 7–9 EGF repeat modules that are highly interactive motifs resulting in downstream activation of Rho GTPases which mediate actin depolymerization, cytoskeletal re‐organization, and cell signaling. A number of brain cell associated and extracellular KS‐PGs have diverse functional roles in the control of neural development, neurotransduction, neural protection, assembly and function of perineuronal nets (PNNs) with roles in cognitive processing, problem solving and memory (Melrose 2019a, 2024b). Instructive cues from the brain ECM (Hayes and Melrose 2021, 2018; Melrose et al. 2021) are operative in the assembly and repair of neural functional networks from components secreted by glial cells and neurons and the formation of transmitter and effector receptors and ion channels that control brain function and neuronal activity (Melrose et al. 2021; Zang et al. 2021). KS directs axonal migration and the assembly of trigeminal nerve growth cones innervating the cornea (Schwend et al. 2012). KS‐PGs have been proposed to act as a template that directs neuroblast migration and axonal fascicular pathways during fetal brain development (Sarnat and Yu 2025). Claustrin and lumican are two KS‐PGs with proposed directional roles in axonal migration during development of the CNS (McCabe and Cole 1992; Burg and Cole 1994). These observations are consistent with multi‐functional instructive properties proposed for KS in the CNS/PNS (Melrose 2025c, 2025d). An alternatively spliced form of MAP1B (microtubule‐associated protein 1B) has an N‐terminal region bearing homology with claustrin (Burg et al. 1997). Claustrin may represent a truncated form of MAP1B; however, claustrin and MAP1B do not share functional identity (Burg and Cole 1994). Claustrin is an anti‐adhesive KS‐PG, MAP1B is highly expressed in neurons and regulates axonal guidance and elongation (Tymanskyj et al. 2012; Bodaleo et al. 2016). Claustrin may also contribute to axonal guidance processes but in a dissimilar manner to MAP1B.

KS interacts with multiple kinases in the brain with important roles in neural development and signaling (Conrad et al. 2010). These interactions occur primarily through KS‐PGs, which are key components of the ECM and cell surface (Melrose 2019a, 2025d). Understanding KS–kinase interactions may be insightful in the elucidation of neuronal regulatory processes and their potential relevance to neurological disorders. The recombinant KS SLRP lumican promotes wound healing through interaction with activin receptor‐like kinase 5 (ALK5) (Yamanaka et al. 2013). ALK5 is a serine/threonine kinase that plays a key role in the TGF‐β signaling pathway acting as a type‐1 TGF β receptor which regulates cell growth, differentiation, and tissue repair.

### 2.5 KS Has Roles in Electrosensory Processes

As a multifunctional, “electro‐sensory” molecule, KS plays a key role in mediating cell‐signaling, tissue structural organization, and, in specific cases, environmental sensing.

KS has important roles in mechanosensory and neurosensory processes, neural signaling, and tissue stabilization (Melrose 2025a, 2024a, 2019a). As a component of diverse cell‐associated and extracellular matrix KS‐PGs, it confers regulatory properties that influence cell behavior and tissue function (Melrose 2019a). KS contributes to mechano‐transduction by converting mechanical forces into biochemical signals that feed into neuro‐transductive regulatory pathways through interaction with a wide range of ligands to mediate cell regulation (Conrad et al. 2010). Beyond its structural roles, KS also participates in electroconductive bioregulation of neural processes (Melrose 2025d). Recent studies highlight the emerging significance of low‐sulfation KS isoforms in novel bioregulatory mechanisms, expanding the functional repertoire of KS‐PGs and their roles in neuro‐regulation in health and disease (Melrose 2025a, 2019a). Historical studies on an electro‐sensory KS glycoconjugate in elasmobranch fish species (sharks and rays) identified a low sulfation KS isoform with sensory capability for the electrical fields generated by prey‐fish species (Melrose 2019b, 2019c; Josberger et al. 2016). Elasmobranch fish species possess an electrosensory organ called the Ampullae of Lorenzini, located mainly in their snouts (De Iuliis and Pulerà 2019). This organ contains a highly sensitive sensory KS glycoconjugate gel that detects protons released by the muscular activity of prey fish species through a process known as electrolocation (Zhang et al. 2018; Josberger et al. 2016). The KS in this gel is a low sulfation isoform, and it is the most sensitive proton detection agent known in nature (Melrose 2025b). Electroreception is shared by primitive aquatic vertebrates and some aquatic amphibians but was lost in amniotes when they transitioned to a terrestrial existence. Two monotreme animals have retained the ability to electrolocate, which is delivered by modified mucous glands in the bill or snout. These are the long and short nose Echidna (spiny anteater, *Tachyglossus aculeatus* ) and Duck‐billed platypus, which use electrolocation for feeding purposes (Proske and Gregory 2003; Scheich et al. 1986; Langner and Scheich 2009).

### 2.6 KS Has Proton Capture and Transfer Properties Operative in Neurosensory Processes

**FIGURE 4:**
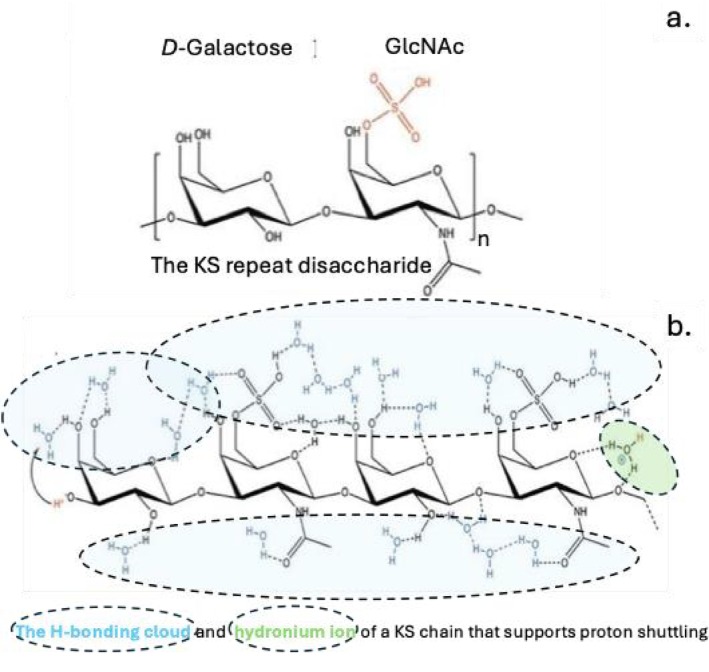
Schematic depiction of the H‐bonding cloud of KS that equips it with proton transfer properties operative in neurotransmission in neurosensory processes.

In the natural world, GAGs conduct protons through the formation of hydrated hydrogen‐bond networks facilitated by their sulfate groups and their hydrophilic nature (Figure 4). GAGs have the ability to absorb water and create proton pathways, a property demonstrated by KS, which is conductive at room temperature under humid conditions (Nagao 2024). Other GAGs like HS, CS, DS, and HA also exhibit this property, but to a far lesser extent than KS, which is the best natural proton conductive polymer so far discovered in nature (Selberg et al. 2019). Protons act like a neurotransmitter (Du et al. 2014), activating postsynaptic acid‐sensing ion channels, which act as proton receptors (Du et al. 2014). This property facilitates synaptic plasticity and is an important requirement in cognitive learning and memory and the functional properties of neural networks (Zeng and Xu 2012). The electroconductive proton capture and transport properties of KS (Josberger et al. 2016; Selberg et al. 2019) facilitate its participation in neuronal electrochemical interactions in neural networks (Deng et al. 2016). This partly explains how KS substituted CNS/PNS ECM proteoglycans provide instructive directional cues in the development and function of neural tissues (Melrose et al. 2021). A central question, however, in the development of complex CNS axonal circuits is how multiple axons are sculpted into balanced functional networks (Kalil and Dent 2014; Chédotal 2019). Lumican, a KS‐PG, has been shown to facilitate inter‐axonal communication in nerve bundles in corticospinal innervation in mice (Itoh et al. 2023). Corticospinal neurons extend axons forming synaptic connections and circuits in the brainstem and spinal cord (Kalil and Dent 2014; Chédotal 2019). These are of functional significance in the development of the control of precise body movements and speech development (Levine et al. 2012; Lemon 2008; Martin 2005).

### 2.7 The Proton Is an Energetic Neuro‐Communicator

The proton is one of the three basic sub‐atomic particles along with neutrons and electrons making up atoms, the building blocks of all matter. Protons are the dominant subatomic particle in the visible universe comprising ≈87% of the particle mass (Suit 2013). Protons not only had fundamental roles in the early evolution of life but also in the emergence of a universal energy production system in mitochondria which powered the evolution of the eukaryotic cell type (Vercellino and Sazanov 2022) and neurosensory processes in health and disease (Collins and Forgac 2020). Neurons are one of the most energy demanding cell types in the human body, and high mitochondrial numbers in neurons are essential to power neuronal synaptic activity in the CNS/PNS (Faria‐Pereira and Morais 2022). Mitochondrial dysfunction is implicated in neurodegeneration in Parkinson's disease, Alzheimer's disease, frontotemporal dementia, Huntington's disease, and amyotrophic lateral sclerosis (Chu 2022).

Four G‐protein coupled receptors (GPCRs), GPR4, 65, 68, and 132, are cellular proton sensors (Cornell et al. 2024). These GPCRs have proposed roles in mechanosensation, inflammation, cancer (Justus et al. 2024), hematopoiesis, inflammatory and neuropathic pain, and cytoskeletal remodeling dynamics in tissue development (Hepler 2016) and in health and disease (Collins and Forgac 2020; Sisignano et al. 2021).

### 2.8 KS‐Proteoglycans Found in the CNS/PNS and in Cartilaginous Tissues

Some examples of KS‐PGs are presented in Table 2 illustrating some of their diverse functions in a range of tissues. KS‐proteoglycans have important roles in the assembly of neural signaling networks which determine the functional properties of the CNS/PNS (Melrose 2019a) and in the assembly of transient rudiment cartilaginous templates which eventually undergo calcification to form the appendicular and axial skeleton (Melrose et al. 2016). KS is also a component of the permanent articular cartilages that line diarthrodial joints with roles in joint articulation (Hayes et al. 2022). Cartilage aggrecan contains a high charge KS rich region and low sulfation KS in the G1, G2 N‐terminal globular domains, and in the inter‐globular domain (IGD). KS chains in the IGD are small *N*‐linked GAGs (Poon et al. 2005), while small *N*‐ and *O*‐linked KS chains are present in the G1 HA binding region (Barry et al. 1995, 1992), KS chains in the KS rich region are highly sulphated and *O*‐linked (Kiani et al. 2002). KS in the KS‐rich region has a high proportion of disulfated disaccharides (55%) and a low proportion (11%) of unsulfated disaccharides, whereas KS in the IGD has less disulfated disaccharides (33%) and a significantly higher proportion of unsulfated disaccharides (33%) (Fosang et al. 2009). The IGD of aggrecan in aged tissues may be more susceptible to proteolysis by aggrecanases due to the presence of low sulfation KS. The low sulfation KS chains in the IGD have been proposed to prime this region for cleavage by aggrecanases (ADAMTS 4, ADAMTS‐5) (Pratta et al. 2000). This is a key region in aggrecan in weight bearing cartilages, IGD cleavage can significantly reduce the aggregation of aggrecan with HA due to expulsion of IGD cleaved aggrecan no longer anchored in the cartilage ECM. This reduces the osmotic pressure exerted by aggrecan in cartilage reducing its weight bearing properties (Mort et al. 2016). HA‐aggrecan macro‐aggregates also promote PNN assembly with brain aggrecan (Morawski, Brückner, et al. 2012), HA also aids in the hydration and compartmentalisation of brain tissues providing specific niches appropriate for optimal neuronal activity (Melrose 2023). Phosphacan also has roles in regulation of the 3D architecture of PNNs affecting synaptic plasticity and cognitive function (Eill et al. 2020). SV2 is a neurotransmitter and storage KS‐PG with roles in neurotransmission (Nowack et al. 2010; Wan et al. 2010; Morgans et al. 2009; Dunn et al. 2017). Several KS substituted SLRPs including lumican, keratocan and fibromodulin have varied roles in the regulation of collagen fiber architecture in the cornea ensuring fine fibril spacing is maintained to ensure corneal transparency and refractile properties essential for vision and large collagen fibrillogenesis in the sclera to provide mechanical stability to the eye.

Podocalyxcin, a 240 kDa sialylated mucin‐like cell transmembrane PG, contains small low sulfation KS chains in embryonic tissues and promotes neurogenesis while in adult tissues and in several tumors podocalyxcin contains highly sulfated KS chains (Le Tran et al. 2021; Toyoda et al. 2017; Vitureira et al. 2010, 2005).

### 2.9 Lumicans' Interactions With the Extracellular Matrix With Roles in Cell Signaling

Lumican has a 37–40 kDa core protein and is a highly interactive PG that contains four potential KS chains and a 10 modular central LRR region in its core protein flanked N and C terminal disulphide stabilized globular domains (Kao et al. 2006; Nikitovic et al. 2008; Chakravarti et al. 1998). N‐terminal sulfated tyrosine residues in lumican contribute to its anionic properties mimicking the ligand interactivity of HS (Kao et al. 2006).

Lumican also has an MMP inhibitor domain, lumcorin, located in LRR9 (Zeltz et al. 2009) and a 13 amino acid C‐terminal cluster (lumikine) which has growth factor‐like activity (Gesteira et al. 2017; Kao et al. 2024).

Lumican supports neural circuit development by organizing the ECM, guiding neuronal connectivity, and integrating specialized neurons. Lumican interacts with ECM components and cell surface receptors to influence cell proliferation, differentiation, and migration (Puttur et al. 2026). By modulating neuronal growth, connectivity, tissue repair, and inflammation, lumican not only provides structural support but also directs cellular behavior essential for neural network development and injury response. Lumican plays a critical role in ECM organization and cell signaling in health and disease (Puttur et al. 2026; Amjadi et al. 2013; Tsui et al. 2023). It regulates collagen fibrillogenesis, contributing to tissue structural integrity, and influences key cellular processes including proliferation, migration, and differentiation (Smith and Melrose 2024). Regulation of thin collagen fibril spacing and 3D architecture is of particular importance in maintaining corneal transparency and its refractive properties in vision (Kao and Liu 2002). LUM interactions with TLR9 and CpG‐DNA indicate a role in immune regulation and inflammatory responses, underscoring its multifunctional involvement in tissue homeostasis and immune signaling (Maiti et al. 2021). A CpG site is a specific location on a DNA strand where a cytosine nucleotide is immediately followed by a guanine nucleotide connected by a phosphate group in the 5′ to 3″ direction. KS is attached to Asn in LUM core protein via a complex‐type N‐linked branched oligosaccharide in four attachment sites (Dunlevy et al. 1998) not all sites may be occupied at one time. Non‐glycanated LUM core protein is widely distributed in the sclera, aorta, cartilage, intervertebral disc, liver, skeletal muscle, kidney, pancreas, brain, placenta, bone, and lung (Johnstone et al. 1993; Ying et al. 1997; Funderburgh et al. 1991, 1993; Krull and Gressner 1992). The non‐glycanated LUM form increases with age due to decreased KS synthesis (J. L. Funderburgh 2002). The presence of KS‐free LUM in tissues may have a significant, but poorly understood, impact on inflammation and disease (Amjadi et al. 2013; Tsui et al. 2023). The lumican core protein, without the KS side chains, is equally efficient in inhibiting in vitro collagen fibrillogenesis, suggesting this function to be entirely core protein mediated (Rada et al. 1993). Addition of KS chains, however, increases Lum's interactive properties with a diverse range of ligands (Conrad et al. 2010) with effects on neural development (Schwend et al. 2012).

#### 2.9.1 Health Promoting Properties of Lumican

Lumican regulates cell proliferation, differentiation, inflammation and the innate and humoral immune systems to combat infection. Lumican regulates macrophage responses to bacterial endotoxin. Lumican associates with CD14 on the surface of macrophages and neutrophils, and promotes CD14‐TLR4 mediated responses to bacterial LPS. The TLR4 signaling pathway detects bacterial LPS initiating an innate immune response regulated by lumican. Primary cultures of (Lum(−/−)) macrophages show impaired innate immune responses to LPS and a reduced induction of TNFα and IL‐6. Supplementation of these cultures with a recombinant lumican core protein increases LPS mediated TNFα induction and partially rescues an innate immune response in macrophages from lumican knock‐out mice. Lumican interacts with CD14 via its N‐terminal sulfated tyrosine residues regulating presentation of bacterial endotoxin to cells. CD14 is a 55‐kDa protein expressed as a glycosylphosphatidylinositol‐anchored membrane protein (mCDI4) and as a soluble serum protein (sCD14) lacking this glycosylphosphatidylinositol anchor (Wright et al. 1990; Pugin et al. 1994; Ulevitch and Tobias 1995; Jiang et al. 2000). CD14 activates cytokine secretion and inflammation in macrophages (Sharygin et al. 2023). Lumican also regulates inflammation in a mouse model of colitis (Lohr et al. 2012) and inflammatory responses in the cornea (Vij et al. 2005; Shao et al. 2013).

#### 2.9.2 Anti‐Tumor Properties of Lumican

A direct link between lumican expression and melanoma progression and metastasis has been demonstrated. Lumican impedes tumor cell migration and invasion by blocking tumor cell interactions with the α2β1 integrin. The LRR9 lumcorin domain of lumican was identified as being responsible for inhibition of melanoma cell migration. Lumican also has angiostatic properties downregulating proteolytic activity associated with endothelial cell membranes, such as MMP‐14 and MMP‐9 (MMP)‐14 and MMP‐9 inhibiting tumor progression (Brezillon et al. 2013). A 17 amino acid peptide derived from LRR‐9 of lumican (lumcorin) is responsible for its anti‐tumor activity and inhibits melanoma progression (Zeltz et al. 2009; Pietraszek et al. 2013). This anti‐tumor activity resides in the ability of lumcorin to inhibit cell motility by inhibition of focal adhesion kinase phosphorylation and by blocking melanoma cell interactions with α2β1 integrin (Zeltz et al. 2009, 2010; D'Onofrio et al. 2008) preventing the development of focal adhesion complexes (Brezillon, Radwanska, et al. 2009) which are required for cell migration. Lumcorin also displays MMP inhibitory activity (Pietraszek et al. 2014) which contributes to the anti‐tumor activity of lumican (Stasiak et al. 2016). Lumican also exerts angiostatic properties on endothelial cells (Niewiarowska et al. 2011) which contribute to tumor inhibitory activity in melanoma and a number of other tumors (Brezillon, Zeltz, et al. 2009).

#### 2.9.3 Roles for Lumican in Diabetes and as a Biomarker of Disease Processes

KS chains bind the insulin‐like growth factor binding protein‐2 (IGFBP2) (Russo et al. 1997) and impair adipocyte metabolism, partially via ERK signaling, and is a potential target for developing adipose tissue‐targeted therapeutics in diabetes (Strieder‐Barboza et al. 2022). Lumican is a potential biomarker of diabetic nephropathy (Tao et al. 2025) and has also been proposed as a biomarker of the hypercoagulative state of long COVID disease (Smith and Melrose 2024).

#### 2.9.4 Lumican and Neurodegenerative Disorders

Unlike connective tissues, the CNS lacks supportive collagen and elastin fiber networks; however, the KS‐PG aggrecan and HA provide structural stabilization (Melrose 2023) especially in PNNs, where besides providing mechanical stabilization, cognitive processes, memory, and synaptic plasticity are also promoted (Otsuka et al. 2025; Carulli et al. 2007). LUM also contributes to brain tissue stabilization and regulates cortical folding during human neocortex development (Long et al. 2018). Following CNS injury, CS and KS‐PGs are upregulated, forming a gliotic scar that stabilizes damaged tissue but inhibits axonal regeneration. SLRPs, including LUM, contribute to this inhibitory environment (Kolb et al. 2023).

LUM plays a key role in axonal guidance during brain development, where it is selectively expressed in the lateral cortex and regulates inter‐axonal communication to ensure correct neural network formation (Itoh et al. 2023). In a 3D BBB model, LUM was the most significantly downregulated SLRP in endothelial cells exposed to altered shear stress reducing barrier integrity. Supplementation with LUM restored BBB function under physiological flow conditions, indicating a critical role for LUM in endothelial cell–matrix interactions and BBB maintenance (Bouhrira et al. 2022). Peripheral endothelial staining shows LUM interacts with cell surface receptors, consistent with its known role in the endothelial glycocalyx (Friden et al. 2011). LUM is also expressed in the spinal cord (Hayes and Melrose 2020) and interacts with inflammatory cytokines such as CXCL1 and receptors involved in cell motility and immune regulation. Disruption of cerebrovascular flow or BBB integrity, as occurs in trauma or stroke, leads to reduced LUM expression and compromised tight junctions. Elevated LUM levels have been detected in cerebral protein deposits in schizophrenia, a disorder associated with abnormal protein turnover and progressive neurodegeneration (Rodrigues‐Amorim et al. 2022).

#### 2.9.5 Lumicans Role in Neuronal Circuit Refinement

In the corticospinal motor system, lumican is secreted by specialized neurons at spinal cord terminals, where it regulates competition between older and newer neuronal connections (Itoh et al. 2023). This bias enables newer, more precise neurons to integrate into existing circuits without disrupting existing networks, thereby refining neural circuits and improving fine motor control, supporting functional specialization and neural plasticity.

### 2.10 Participation of KS in Neurosensory Processes

The ability to perceive ion‐fluxes by KS is a conduit that provides sensory capability to cells allowing them to perceive their environment and to respond dynamically to biomechanically mediated changes in their microenvironment equipping them with the ability to modulate the synthesis and assembly of ECM components to form a matrix which better protects them from extrinsic forces. In some cells, the ability to sense and control ion‐fluxes is highly advanced such as in neurons which generate action potentials (Camire and Topolnik 2014), the basis of synaptic function and has even been proposed as a Ca^2+^ flux type mechanism whereby cognitive memory is generated (Eccles 1983).

#### 2.10.1 KS and Audition

KS plays a key role in auditory neurosensory function within the cochlea of the inner ear in mammals. The cochlea is a fluid‐filled, spiral structure containing the organ of Corti, which transduces sound‐induced fluid displacement into neurosensory signals which are transmitted to the brain (Goodyear and Richardson 2018). Sound wave‐generated fluid displacement is detected by the tectorial membrane in the cochlea (Sellon et al. 2019; Richardson et al. 2008; Gavara et al. 2011). This signal is conveyed to adjacent sensory hair cells connected to the auditory nerve network (Goodyear and Richardson 2018; Fettiplace and Kim 2014; Fettiplace 2017; Fettiplace and Hackney 2006). Highly sulfated KS is concentrated on the tectorial membrane surface and on hair cell tips (Killick and Richardson 1997; Katori et al. 1996), whereas low‐sulfated KS is found on hair shaft membranes and within the tectorial membrane matrix (Melrose 2024a). This mechanism parallels sensory processes in fish neuromast cells of the lateral line system, which detect water movement and pressure changes (van Netten and McHenry 2013). Canal neuromasts sense fluid flow acceleration through dense clusters of hair cells, while superficial neuromasts detect flow velocity, enabling perception of subtle water movements in low‐visibility environments (Bleckmann and Zelick 2009). Similarly, sound‐induced fluid displacement in the cochlea underlies auditory perception in mammals. The precise way KS undertakes these sensory auditory processes needs further investigation but nevertheless this provides evidence of the involvement of KS in higher neurosensory processes.

##### 2.10.1.1 Use of KS Knock‐Out Studies to Delineate the Roles of KS in Auditory Processes

The immunolocalization of KS in specific regions of the cochlea (Munyer and Schulte 1994, 1995) is an interesting observation; however, these are essentially anecdotal in nature and need to be followed up with functional studies on KS, possibly using KS knockout models to precisely define the function of KS in audition. Recent advances in the elucidation of the functional properties of GAGs including KS in the regulation of CNS/PNS neurosensory processes indicate similar studies are warranted in the cochlea (Schwartz and Domowicz 2023; Melrose 2024a, 2019a, 2019b; Smith et al. 2015). The recent development of single cell RNA seq imaging of cochlear tissues has significantly improved resolution of the cellular complexity in the cochlea (Burns et al. 2015). Functional MRI has also been used to assess the functional inter‐connectivity of neural and matrix components (Mitchell et al. 2026; Giubergia et al. 2026). These methods could be applied in KS knockout models, such as the β‐1,3‐N‐acetylglucosaminyltransferase‐7 (β3GnT7)‐null mouse model, which has delineated the role of KS in corneal tissues (Littlechild et al. 2018).

In tensional and weight bearing connective tissues KS occurs as a side chain component of PGs such as aggrecan which provides mechanical support and in PGs which regulate collagen fibrillogenesis which is also mechanically supportive, thus KS supports mechanical properties in tissues. When these PGs are damaged this leads to the emergence of diseases in soft connective tissues (Halper 2014; Berdiaki et al. 2024). KS‐PGs also have diverse roles in the assembly and function of neural tissues. The observation of KS localisations in the tectorial membrane and the tips of stereocilia in sensory hair bundles suggests KS may have mechanical roles to play in these tissues.

The stereocilia of the sensory hair bundle, a mechanosensitive organelle, are composed of elaborately arranged actin microfibrillar arrays arranged in rows of increasing height. It is remarkable that sensory hair cells can detect hair bundle deflections smaller than 1 nm showing the extreme sensitivity and precision of this signaling apparatus (Vollrath et al. 2007). Extracellular and internal linkages between hair cells in hair bundles support cohesion and the conveyance of forces from the tectorial membrane which are channeled through mechanically gated channels at the tip of stereocilia generating mechano‐electro‐neurosensory signals which are transferred by neural networks to the brain for processing of auditory signals. Lactosamine glycoconjugates have been immunolocalised in the tectorial membrane and sensory epithelia of the organ of Corti (Hozawa et al. 1993). It has been suggested that these may have some role in the conversion of sound waves to nerve impulses aiding in audition. Highly sulfated KS is present on the surface of the tectorial membrane and the tip of stereocilia while low sulfation KS occurs in the mid‐substance of the tectorial membrane and along the shafts of stereocilia (Munyer and Schulte 1994, 1995). The significance of this observation in the cochlea has yet to be specifically determined but bioregulatory roles for low sulfation KS are emerging in connective tissues (Melrose 2025a).

##### 2.10.1.2 KS Deficiency in Auto‐Immune Neuritis Effects Neural Degenerative Pathology

KS is associated with activated microglia/macrophages that accumulate in neuronal injuries, KS expression; however, is diminished in experimental autoimmune neuritis (Matsui et al. 2013). Silencing of KSGal6ST attenuates KS expression in primary microglial cultures and upregulates expression of inflammatory cytokines (TNF‐α, IL‐1β, and iNOS) contributing to the degenerative pathology seen in neurodegenerative disorders such as autoimmune neuritis.

#### 2.10.2 KS and Mechanoreception

**FIGURE 5:**
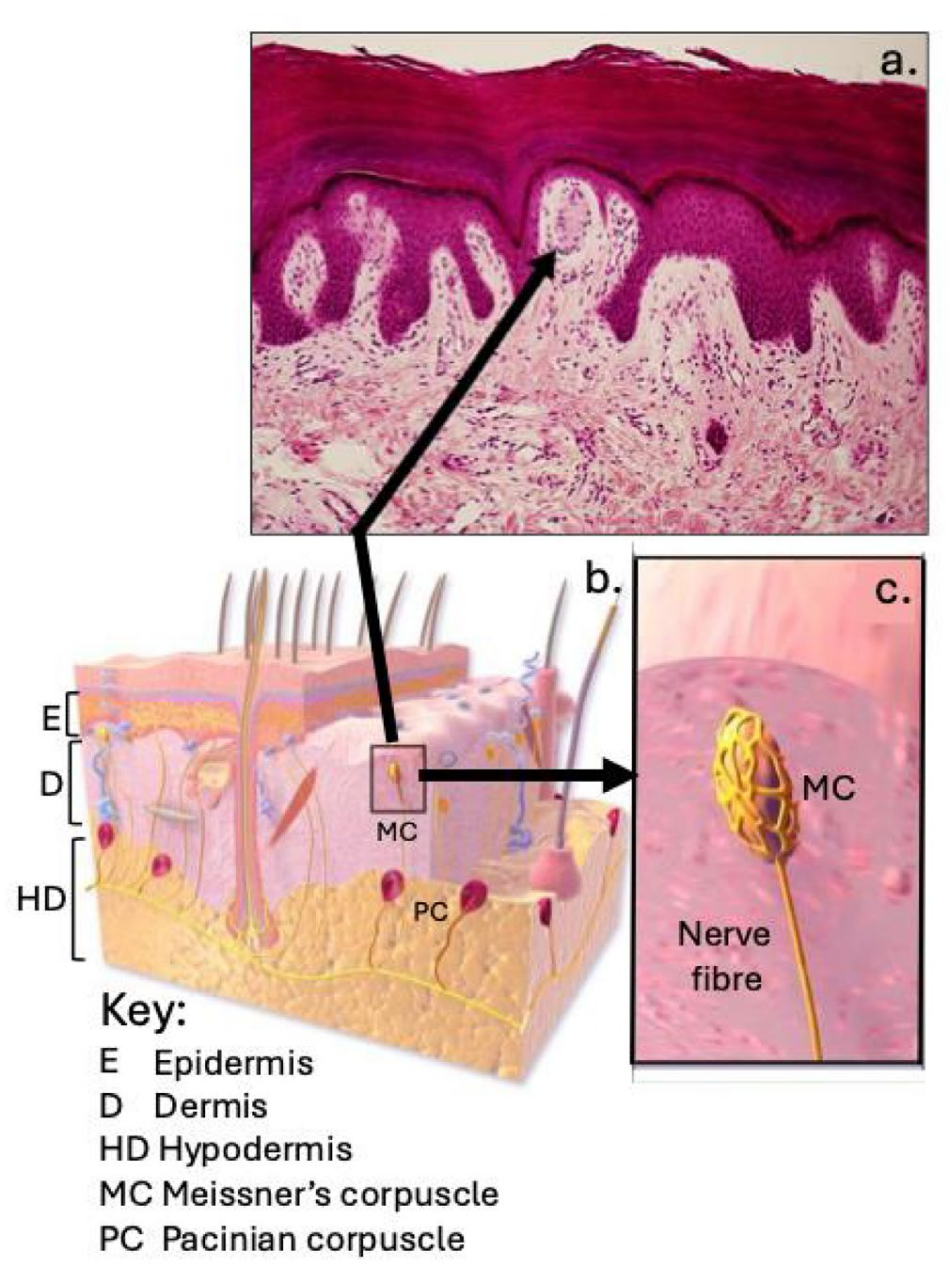
Composite figure depicting histochemical localization of the stratified cornified epithelium, epidermis and dermal layers and location of Meissner's corpuscle in the dermis adjacent to the epidermis. Segment (a) reproduced from Whitehead and Grider (2026). Segments (b, c) reproduced from Piccinini et al. (2025) using OpenAccess.

**FIGURE 6:**
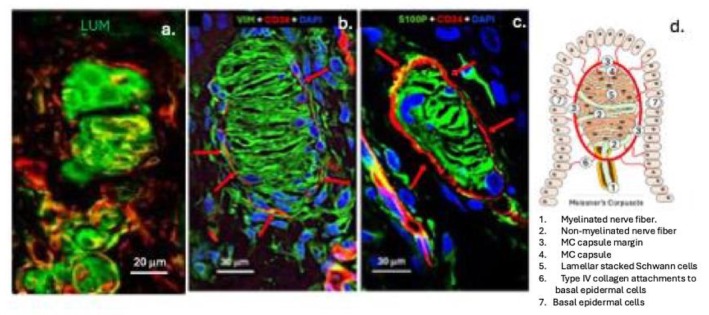
Immunolocalisation of Lumican (LUM), Vimentin (VIM), S100P and CD34 in Meissner's corpuscle (MC) (a, b) and the structural organization of Meissners corpuscles (MCs) shown diagramatically (c). Labeled MC components (d). Vimentin clearly delineates the stacked lamellar Schwann cells in MCs and shows the tortuous pathway a central nerve fiber must navigate through the capsule. Type IV collagen is also a component of the MC capsule and colocalizes with S100P delineating the margins of the capsule along with CD34. Lumican is also a matrix component (not shown) (b). The nerve fiber entering the MC capsule is myelinated (1), but once inside the capsule, it is unmyelinated (2). Images (a) and (b) reproduced from García‐Piqueras et al. (2020) using Open Access.

The KS SLRP lumican has been immunolocalised in Meissner's corpuscles which are encapsulated mechanoreceptors located in the dermal papillae of glabrous (hair‐less) skin (Figure 3) and are responsible for detecting fine touch and low‐frequency vibration (Iheanacho and Vellipuram 2025; Pasterkamp 1999). Structurally, they consist of flattened Schwann cells arranged in layered lamellae, with an un‐myelinated nerve fiber which takes a tortuous course between them along the length of the corpuscle. Meissner's corpuscles measure approximately 30–140 μm in length and 40–60 μm in diameter; these receptors are particularly concentrated in highly sensitive regions such as the fingertips and lips, where they support precise tactile discrimination (Pasterkamp 1999). The mechanoreceptor proteins Piezo‐2, ASIC 1–5, TRVP‐1‐3,4,6 (Holzer 2009; Sherwood et al. 2012; Chen and Wong 2013; Woo et al. 2015) and the KS SLRP lumican have been immunolocalised in Meissner's corpuscles which is consistent with roles for mechanoreceptor proteins in mechanoreceptors; however, the role of lumican has not been determined (Iheanacho and Vellipuram 2025). These mechanoreceptor proteins aid in sensory functions in the detection of touch, pressure, vibration and stretch, with mechanical forces being converted into electrical signals that are transferred to the CNS by a central nerve fiber (Wang et al. 2015; Handler and Ginty 2021; Abraira and Ginty 2013) (Figures 5 and 6). Lumican is a multifunctional proteoglycan; its KS chains have electroconductive properties in neurotransmission (Melrose 2025d) aiding in tactile signal transmission in Meissner's corpuscles (Cobo et al. 2020).

##### 2.10.2.1 KS Has Roles in Mechano‐Electro‐Transduction and Audition in the Cochlea

**FIGURE 7:**
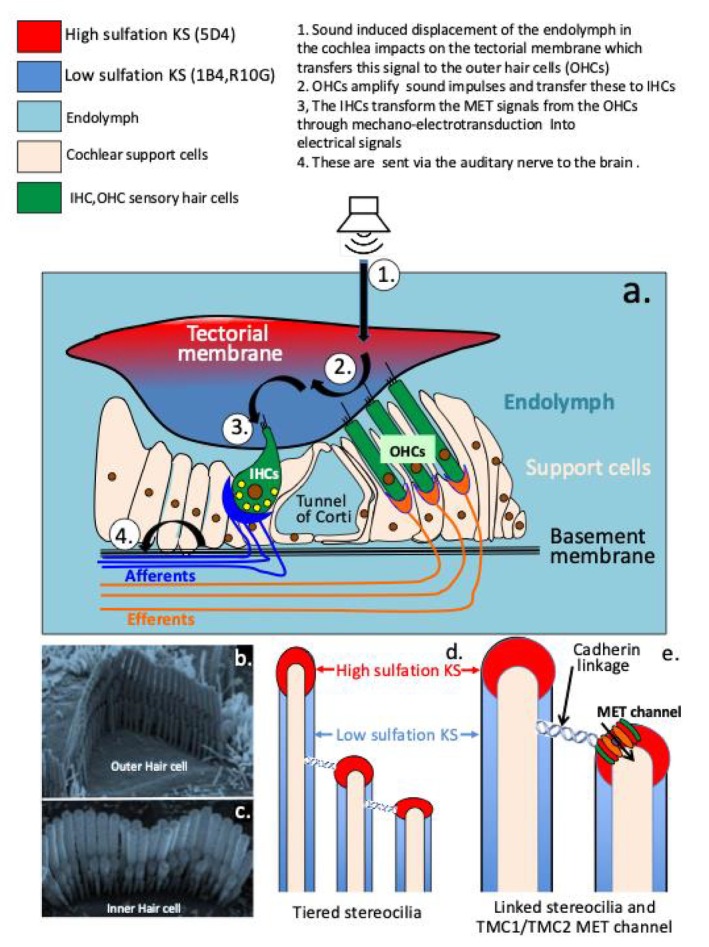
Schematic depiction of sensory structures that transfer sound impulses via the tectorial membrane and stereocilia of the outer and inner hair cells (OHCs and IHCs) and auditory nerve to the brain to facilitate audition in the cochlea.

Small (1–100 nm) movements of the hair‐cell stereociliary bundles induced by sound displacements transmitted from the cochlea tectorial membrane are transmitted via interciliary tip links to open mechano‐electro‐transduction (MET) channels located on the tops of the stereocilia (Fettiplace and Kim 2014; Cunningham and Müller 2019). High sulfation 5D4 positive KS has also been immunolocalised on the stereocilia tip; however, its roles in the MET process still have to be defined. The membranes along the stereocilia hair shafts contain low sulfation KS with unknown functions. The surface of the tectorial membrane also contains 5D4 +ve KS, but its inner regions contain a low sulfation KS isoform (Swartz and Santi 1997; Hultcrantz and Bagger‐Sjoback 1996) (Figure 7). The mammalian transmembrane channel‐like proteins 1 and 2 (TMC1 and TMC2) are components of the MET channel complex (Kurima et al. 2015). These have emerged as promising candidates aiding in MET in hair cells. TMC1/2 have pore‐forming roles in ion channels operative in MET (Fu et al. 2025). It has been suggested that MET is Ca^2+^ dependent (Hakizimana 2024). TMC1 and TMC2 are key components of the MET channel at the tip of stereocilia. Mechanotransduction mediated through the MET channel in the specialized stereocilia of cochlea hair cells is fundamental to the perception of sound waves (Kurima et al. 2015). This intricate mechanism converts mechanical vibrations into electrical signals that the brain can interpret as sound (Deng and Yan 2025; George and Ricci 2026). Proteomics has been used to identify components of the hair‐cell interactome involved in cochlear sound amplification (Zheng et al. 2009). The OHC motor protein, prestin, appears to be associated with electron transport proteins. Prestin confers electromotility properties to sensory hair cells (Zheng et al. 2022) amplifying their movements within the organ of Corti, supporting sensorineural sound amplification (Dallos et al. 2006). Prestin is a motor protein of the OHCs essential for sensitive hearing but is not expressed by non‐motile IHCs. Electrochemical proton gradients in mitochondrial energy production also involve electron transport proteins and have been linked to neurosensory processes (Melrose 2026). The neuron is one of the most energy demanding cell types in the human body. While all GAGs have proton capture and transport properties, KS is the most electroconductive GAG and is the most potent proton capture agent known in nature (Melrose 2024a, 2019b). KS promotes neuro‐transduction, allowing it to promote neural signaling processes in the absence of neurotransmitters (Melrose 2025b) (Figure 7).

##### 2.10.2.2 KS‐Proteoglycans Regulate Corneal Transparency and Visual Function

**FIGURE 8:**
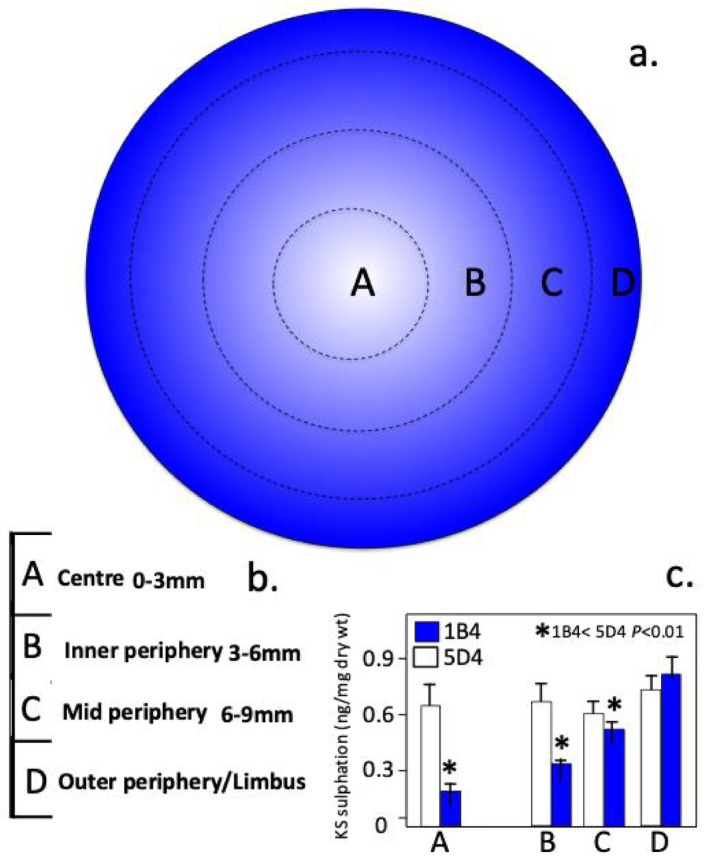
Differential distribution of the low sulfation KS isoform in the mammalian cornea (a) and zonal analysis (b) of high KS sulfation and low sulfation KS isoforms detected using the monoclonal antibodies 5D4 and 1B4 (c). While 1B4 KS shows a differential distribution in corneal tissues, high sulfation KS detected with monoclonal antibody 5D4 shows more uniform high expression levels in all corneal zones examined. These differing distributions of high and low sulfation KS isoforms were statistically significant (*). Figure modified from Melrose (2025d) Open Access.

The cornea has a crucial role in light refraction, contributing over 70% of the eye's total refractive power essential for visual processes. Lumican has essential roles in the control of thin collagen fiber 3D architecture that ensures the optical properties of the cornea are maintained (Tsui et al. 2023). Lumican also promotes repair processes in the cornea in disease (Kao et al. 2024). Lumican has specific functions that protect ocular tissues against biological and chemical damage, as well as providing biomechanical stabilization and structural resilience (Kao and Liu 2002; Blackburn et al. 2019; Chowdhury and Shah 2021). Lumican has pivotal roles in the maintenance of physiological tissue homeostasis and is often upregulated in pathological conditions, for example, fibrosis, scar tissue formation occurring with tissue injury, and in inflammatory responses in disease (Saika et al. 2000). KS occurs as low and high sulfation isoforms which are spatially distributed in gradients in the cornea (Figure 8). Lumican regulates 3D collagen fibril architecture in the cornea ensuring corneal clarity and refractive properties are maintained conducive to visual processing (Chakravarti et al. 1998). Lumican knockout mice develop opaque corneas with aging demonstrating the important roles for lumican invisual processes (Song et al. 2003).

## 3 Concluding Remarks

### 3.1 A Summary of the Functional Attributes of KS

#### 3.1.1 Electroconductive and Proton‐Transport Properties

KS has exceptional proton capture and transport capability (via Grotthuss‐type proton shuttling), helping stabilize electrochemical gradients and ion fluxes at neuronal membranes. This supports neuronal activation and neurotransduction, even in the absence of classical neurotransmitters. Findings uncovered so far suggest may act as a functional neural interface.

#### 3.1.2 Key Roles in Neurosensory Processes

KS is a prominent component of neurosensory tissues, including the cochlea, mechanoreceptors (e.g., Meissner's corpuscles), spinal cord, and electrosensory organs in some vertebrates, where it contributes to mechano‐electro‐transduction and sensory signal propagation.

#### 3.1.3 High Ligand and Protein Interactivity

Highly sulfated KS interacts with a broad range of proteins, including kinases, growth factors, axonal guidance molecules (Eph/ephrins, Slit–Robo), cytoskeletal proteins, and neuro‐regulatory ligands, enabling fine control of neuronal signaling and development.

#### 3.1.4 Context‐Dependent Regulation of Axonal Growth

KS can act as either an inhibitory or permissive cue for neurite extension, depending on sulfation pattern and tissue context. This duality in function facilitates precise neural circuit formation and may be potentially exploitable for guided nerve regeneration.

#### 3.1.5 Structural and Protective Roles in Specialized Tissues

In tissues such as the cornea and perineuronal nets, KS‐PGs maintain hydration, structural organization, mechanical stability, and, in the cornea, optical transparency.

## 4 Conclusions

This review has shown that KS is widely distributed across tissues as a side‐chain component of a range of ECM and cell‐associated PGs. KS‐PGs help regulate connective tissue, stabilize load‐bearing areas, influence cell activity, neural conductivity, and guide connective tissue and neural network assembly. In the cornea, KS‐PGs are essential for maintaining corneal transparency supporting visual function. KS is also prominent in neurosensory tissues, where it may participate in neurotransmission and mechano‐ and electro‐transductive processes in audition. These functions await full elucidation. While a lot still needs to be learned about KS and its functional properties, information gleaned so far points to KS having fascinating properties that deserve further elucidation.

## Declaration of Transparency

The authors, reviewers and editors affirm that in accordance to the policies set by the Journal of Neuroscience Research, this manuscript presents an accurate and transparent account of the study being reported and that all critical details describing the methods and results are present.

## Author Contributions

J.M. conceptualized and wrote this manuscript in its entirety including preparation of figures and revision of the manuscript to its final form.

## Funding

This work was funded by The Melrose Personal Research Fund, Sydney, Australia.

## Disclosure

J.M. has received consultancy fees from Arthropharm‐Fidia Pharmaceuticals, Sydney. This company had no input into the writing of the manuscript or interpretation of data.

## Conflicts of Interest

The author declares no conflicts of interest.

## Supporting information


**Data S1:** Transparent Science Questionnaire for Authors.

## Data Availability

All data necessary to evaluate the conclusions of this manuscript are provided in the main text.
